# Effects of Music Choice on Performance and Psychophysiological Responses to Exercise—A Scoping Review

**DOI:** 10.3390/jfmk11020144

**Published:** 2026-03-31

**Authors:** Emily S. Pounds, Scott W. Snyder, Rebecca R. Billings, Haley M. Nguyen, Christopher G. Ballmann

**Affiliations:** 1 Department of Physical Therapy, University of Alabama at Birmingham, Birmingham, AL 35294, USA; emilysg@uab.edu (E.S.P.); nguyenhm@uab.edu (H.M.N.); 2 Department of Human Studies, University of Alabama at Birmingham, Birmingham, AL 35294, USA; ssnyder@uab.edu; 3 Univeristy of Alabama at Birmingham Libraries, University of Alabama at Birmingham, Birmingham, AL 35294, USA; beccarb@uab.edu

**Keywords:** self-selected music, performance, motivation, psychological, neuromuscular

## Abstract

Listening to music is a well-established strategy to enhance exercise capacity, yet the specific mechanisms linking music choice to performance enhancement remain fragmented. This scoping review systematically summarizes the existing literature on the effects of music choice (i.e., self-selected, preferred music) on performance and psychophysiological determinants of exercise capacity to establish an updated rationale for the use of personalized music interventions in training. Following PRISMA-ScR guidelines, a systematic search of five databases (PubMed, Web of Science, Embase, Scopus, CINAHL) was performed for studies published between January 2000 and April 2025. Peer-reviewed articles investigating the ergogenic effects of self-selected or preferred music with psychophysiological outcomes were included. Thirty-two studies met inclusion criteria. Overall, evidence supports consistent performance enhancement from choice music (CM) across modes of exercise including aerobic endurance, anaerobic power, and muscular endurance activities while maximal strength was largely unaffected. The most robust and consistent mechanisms underpinning the benefits of CM during exercise were psychological in nature, including improvements in affect, arousal, motivation, and perception of exertion. Notable physiologic benefits were also identified, including altered cortical excitability, autonomic modulation, and improvements in neuromuscular efficiency. Herein, this review provides a psychophysiological framework whereby CM acts as a primary mediator to induce psychological and physiological cascades which synergistically contribute to ergogenic benefits. Evidence heavily supports the superiority of CM to improve exercise performance across various modalities.

## 1. Introduction

The use of music during exercise is widely utilized by athletes and recreationally active individuals due to its ergogenic properties. Indeed, historical accounts and observations dating back centuries, and possibly millennia, document the link between rhythm, movement, and alterations in emotional or psychological states [1]. In modern day sports, listening to music has been reported to result in performance enhancement across a wide range of exercise modalities including aerobic endurance, resistance-based, anaerobic sprint, and interval-based exercises [2,3,4,5,6]. However, the degree of music-induced benefit has been repeatedly linked to the listener’s choice (i.e., self-selection, preference), suggesting the need for individualized approaches to optimize ergogenic effects beyond merely the presence of sound [2,7,8,9].

Across the literature, a central theme of music-induced exercise benefits is a modulation of key psychological and physiological mechanisms mediating performance. Importantly, choice music (CM), or music that is personally chosen by the individual exercising, has been established to impart distinct advantages by more potently altering psychophysiological processes versus pre-determined (i.e., non-self-selected music) or non-preferred music. Psychologically, CM has been shown to enhance psychological mediators of exercise performance including affective valence, motivational state, and psychological arousal [2,10,11,12]. CM has also been suggested to result in deeper levels of attentional shift and dissociation, thereby potently modifying perception of discomfort and exertion [13,14]. Taken together, CM leads to psychological improvements in readiness to give effort, exercise enjoyment, and diverts focus from feelings of fatigue [2,14,15]. Physiologically, CM primarily alters various neurophysiological processes and can selectively influence activation of both central and peripheral mechanisms. Evidence utilizing neuroimaging techniques has demonstrated that brain regions responsible for arousal, emotional regulation, and motivation are potently influenced by CM [16,17,18,19]. Furthermore, alterations to cortical excitability, autonomic control, and sensory integration have been observed with CM [20,21,22]. For example, electroencephalographic (EEG) measurements during warm-ups before exercise showed that CM lowers the theta-to-beta ratio, likely reflecting a heightening of arousal or “psyching up” effect [20]. Thus, CM-induced benefits are likely a result from concomitant changes in psychological and physiological processes which culminate in performance enhancement.

Despite this, the purported effects of music in the literature are not homogenous, and identification of the principal mechanisms underlying performance enhancement with music are fragmented. This is likely a consequence of different exercise modalities, measurement techniques, timing of music application, and degree of music choice. Consequently, a cohesive model explaining psychological and physiological underpinnings of listening to CM is lacking. Furthermore, previous reviews related to CM and exercise performance were not systematic in nature and may be prone to unintentional omittance of key evidence and bias [2]. Therefore, the objectives of this scoping review were to: (1) systematically summarize the existing literature to identify the distinct psychophysiological mechanisms that mediate the ergogenic effects of CM on exercise performance in healthy populations, (2) provide in-depth discussion of the effects of CM on anaerobic and aerobic-based exercise performance, and (3) use the existing literature to form a cohesive psychophysiological framework for the effects of CM on exercise performance outcomes. This review specifically encompasses both self-selected and preferred music as CM and explores underlying mechanisms for performance enhancement, in both physiological and psychological domains. Disparities and similarities between findings are highlighted and discussion of limitations, challenges, and future research on CM research needs are also included.

## 2. Materials and Methods

### 2.1. Protocol and Literature Search

This scoping review was conducted and reported in accordance with the Preferred Reporting Items for Systematic reviews and Meta-Analyses extension for Scoping Reviews (PRISMA-ScR) guidelines [23,24]. This scoping review protocol was registered on the Open Science Framework (Registration: https://osf.io/5897k; 26 Feburary 2026). A comprehensive search of five electronic databases was conducted for studies published between 1 January 2000 and 30 April 2025: Web of Science, PubMed, Embase, Scopus, and CINAHL. The search strategies combined keywords and controlled vocabulary related to CM selection, preference, exercise, performance, and psychophysiological outcomes. Given the heterogeneity of nomenclature, both self-selected and preferred music conditions were considered CM, while conditions of pre-determined (i.e., researcher chosen) and non-preferred music were considered contrasts. Silence or no music (NM) was also reported as a condition by some studies. The search was limited to articles published in English and involving human participants. No supplementary search methods such as citation searching or screening of gray literature were performed. The full, detailed search strings for each database are provided in (File S1).

### 2.2. Eligibility Criteria, Search, and Review of Evidence

To be included, studies had to be peer-reviewed articles that investigated the effects of self-selected or preferred music compared to conditions of non-preferred music, pre-determined music, or silence in healthy physically active adults. Furthermore, included studies were required to have a distinct exercise component and measure at least one performance task outcome (e.g., speed, endurance, power) and a psychophysiological outcome (e.g., RPE, heart rate, motivation). Studies were excluded if they lacked an exercise component, were theoretical papers or literature reviews, or employed factorial interventions. This exclusion allowed the review to focus strictly on CM as the central intervention, rather than analyzing it alongside other variables. Further exclusions were applied to studies focused on populations with incorrect health status (i.e., chronic disease).

**Figure 1:**
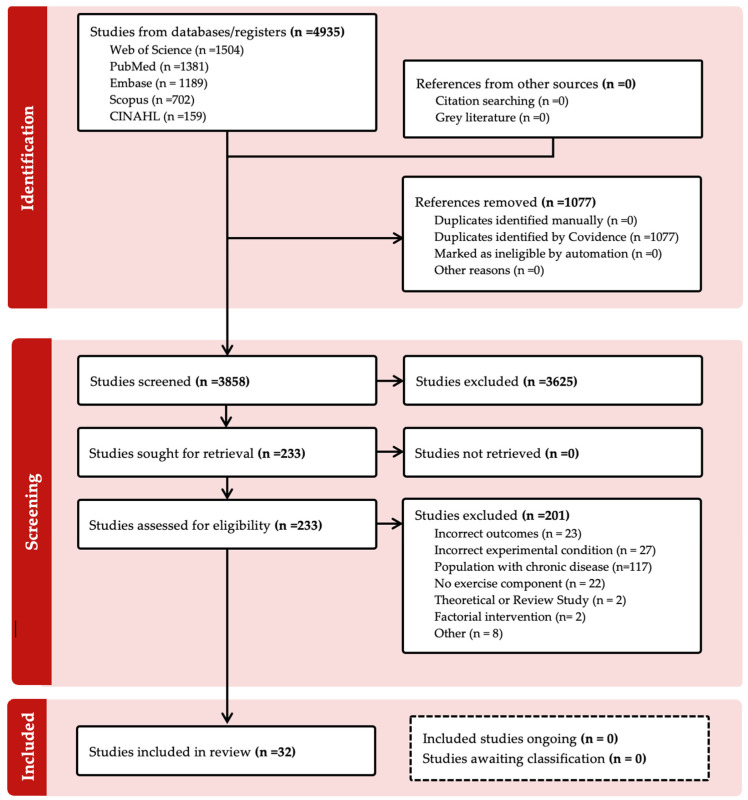
PRISMA 2020 flow diagram for new systematic reviews which included searches of databases and registers only.

The initial database search yielded 4935 records (Figure 1). After these records were imported into Covidence (Covidence systematic review software, 2026, Veritas Health Innovation, Melbourne, Australia. Available at www.covidence.org), 1077 duplicates were removed, leaving 3858 unique studies for title and abstract screening. Two reviewers independently screened all titles and abstracts, after which 233 studies were assessed for eligibility via full-text review. During this review, 201 studies were excluded for reasons such as having no exercise component (n = 22) or focusing on populations with chronic disease (n = 117). Disagreements between reviewers were resolved through consensus or by a third reviewer. Ultimately, 32 studies met all inclusion criteria and were included in this scoping review. Two reviewers independently extracted data from the included studies into a standardized charting form. Variables extracted included: study characteristics (author, year, design), participant demographics (sample size, sex, age, training status), exercise modality, music intervention details, performance outcomes (e.g., speed, endurance, power, task accuracy), and psychophysiological measures (e.g., RPE, heart rate, motivation, affect).

Extracted data were tabulated, and a narrative synthesis was conducted. Results were first organized by exercise modality, categorized as either aerobic or anaerobic. For this review, exercise tasks lasting longer than two minutes were classified as aerobic [25]. Within each modality, findings were further grouped into performance outcomes and psychophysiological outcomes to identify common patterns and knowledge gaps.

## 3. Results

### 3.1. Study and Participant Characteristics

Of the 32 included studies, publication dates ranged from 2013 to 2025. The participants recruited across the 32 studies generally consisted of young, healthy, and physically active individuals. Across the 32 studies, 18 recruited exclusively male participants (e.g., [9,26]), 2 included only females (e.g., [15,27]), and the remaining 12 utilized mixed-sex samples (e.g., [28,29]). The mean sample size across all studies was n = 19 and ranged from an n = 10 to n = 60.

### 3.2. Music Conditions

Included studies compared self-selected and preferred music conditions to pre-determined (i.e., researcher chosen), non-preferred, or no music conditions. Of all studies reviewed, 18 out of 32 (56%) included a self-selected music condition while 14 out of 32 (43%) included a preferred music condition. In total, 10 out of 32 studies (31%) reported non-preferred music conditions and 25 of 32 studies (78%) included a no music condition. A commercial playlist was used by 1 of 32 (3%) studies. In terms of timing of music application, the majority of investigations applied music conditions during exercise (82%). However, 6 of 32 studies (18%) employed music conditions during a warm-up or pre-task.

### 3.3. Exercise Task/Modality

The 32 studies were categorized based on exercise modality to map the primary application of CM. Aerobic Focus (Table 1): The review included 13 studies dedicated to aerobic endurance exercise (e.g., continuous running, cycling time trials, or time to exhaustion tests). Anaerobic Focus (Table 2): The remaining 19 studies focused on analyzing anaerobic exercise (e.g., resistance training, strength, sprints, and HIIT protocols). This distribution confirms that there is comparatively greater attention paid to the effects of music on anaerobic performance, indicating a strong research emphasis on using music to enhance immediate force output and muscular performance metrics (e.g., repetitions to failure).

### 3.4. Primary Outcomes

The synthesized literature consistently approached the CM intervention with a dual focus on both objective and subjective measures of performance and psychophysiological responses to exercise. For aerobic/endurance exercise studies, the most commonly reported performance outcomes were exercise time, distance accumulated, and/or speed while anaerobic studies more commonly reported power output, repetition volume, and/or muscular strength. Across all studies, including endurance and anaerobic, objective measures of exercise intensity were largely reported via heart rate with blood lactate being more focused in aerobic/endurance exercise studies. The most commonly reported subjective outcomes across all studies included RPE, motivation, and varying aspects of affect (i.e., arousal, enjoyment). Advanced objective measures of neuromuscular and cortical activity (e.g., EMG, EEG) were rare, appearing in only two recent studies [20,42].

## 4. Discussion

### 4.1. Aerobic/Endurance Exercise

Current findings from this scoping review suggest that CM potently influences both performance and psychophysiological responses to endurance exercise although this is contextually dependent on modality of exercise, intensity of the activity, and task structure. Across exercise paradigms, cycling and running (≥2 min of continuous activity) were the most common modalities and show that CM most consistently affected total distance covered, time to completion, and self-regulated intensity. Regarding psychophysiological outcomes, RPE, heart rate, blood lactate, and motivation were most commonly reported but showed more heterogenous responses.

Of the 13 studies included that measured indicators of endurance performance, 10 studies (77%) reported enhancement of at least one endurance performance outcome. For example, Nakamura et al. showed that listening to CM resulted in greater cycling distance in recreationally active cyclists during a critical power test [7]. Further bolstering this, Jebabli et al. reported that CM enhanced running speed and distance covered in recreationally active individuals [26,34]. Labudavic et al. also showed improvements in time to exhaustion during graded exercise treadmill tests [27]. Importantly, the ergogenic benefits of CM appear to transcend exercise modality as performance enhancement was shown in endurance running, rowing, cycling, and high-intensity interval exercise [26,27,29,31,36]. Although the links to performance enhancement across studies are not fully clear, it is plausible that CM enhances performance through pacing. Indeed, previous evidence has suggested that synchronizing movement to the rhythm or tempo of music may improve exercise efficiency while asynchronous may show less benefit [53,54]. Mechanistically, this is also supported by evidence showing musical rhythm may result in neural entrainment and enhanced activation of sensorimotor and cortical brain regions [55]. Although speculative at this time, it is possible that music selected or preferred by participants may result in more potent neural entrainment. Indeed, previous work has shown that CM may enhance corticospinal excitability and sensory motor rhythmic integration [53,54]. While these findings largely support the ergogenic effects of CM during endurance-based exercise, disparities still exist. Multiple investigations have shown no ergogenic benefit during cycling or running in well trained individuals [32,36]. While not confirmed, these dissimilarities in findings may be due to differences in training status of participants. Null findings may be due to ceiling effects in highly trained participants, as pacing effects or changes in attentional focus from CM may have limited benefits in populations where high-intensity or familiar tasks are habitually experienced. This is supported by previous investigations showing that untrained individuals may benefit more from music during exercise compared to trained counterparts [56]. However, the current review was not able to dissect potential differences in efficacy between differing training statuses, suggesting the need for additional controlled trials where training and music are at the forefront of testing.

Alterations in psychophysiological outcomes were also widely supported albeit were more variable in which particular outcome was altered. Out of the 12 studies reporting findings with endurance exercise modalities, 9 (75%) reported changes in at least one psychophysiological outcome. In particular, outcomes related to emotion or motivation appeared to be the most frequently observed. Hutchison et al. showed that CM resulted in enhanced feelings of pleasure and enjoyment during self-selected intensity running [33]. Similarly, Lane et al. showed that CM resulting in increased pleasant and decreased negative emotions during running exercise in healthy runners [35]. Karow et al. also showed improvements in feelings of motivation with CM during a warm-up with endurance rowing exercise [29]. Although not directly observed from reviewed studies, improvements in emotional and motivational state are likely a consequence of CM-induced modulation of reward pathways which may result in improved affect and motivational state [16,17]. Improved emotional state and motivation may lead to greater effort allocation resulting in improved performance. This is further supported by previous investigations showing that neural pathways important for reward and emotional regulation are more potently engaged by CM [57]. Furthermore, reductions in RPE were reported by multiple investigations supporting previous reports that showed lowering of RPE as an important psychophysiological mechanism underlying CM-induced changes in exercise performance [7,27,28,37]. CM has been suggested to result in enhancement of dissociation thereby diverting attention away from the discomfort of exercise [2]. While the precise mechanisms underpinning changes in RPE with CM are not fully clear, neuroimaging studies have shown concomitant activation of brain regions important for perception of external focus and fatigue. Indeed, CM has been shown to alter pre-frontal cortex activation, specifically the inferior frontal gyrus, which was negatively correlated with perceived exertion during an isometric task [58]. However, some studies showed no change or even an increase in RPE during endurance exercise with CM [26,32,34]. The observed disparities may be due to differences in timing of the application of CM interventions or duration of exercise. For example, Karow et al. showed that CM played only during a warm-up did not modify RPE during a rowing time trial [29]. It is possible that since CM was not played during the actual exercise bout, there was no external stimuli to cause attentional shifts from the discomfort of exercise, thereby mitigating any changes in RPE. Also, Jebabli et al. showed no differences in RPE during 6 min run tests in runners and physically active males [26,34]. RPE may have remained unchanged due to the shorter duration of the exercise although this is not fully supported as some studies have suggested music may impart dissociative benefits early in exercise [37]. Regardless, RPE seems to be responsive to CM during endurance exercise in some cases but differences in methodology and approach between studies make the translation of findings to definitive conclusions difficult.

### 4.2. Anaerobic/Resistance Exercise

Overall, the effects of CM on anaerobic/resistance exercise have been widely established and comparatively more well described than endurance exercise. CM-induced ergogenic effects have been shown across various modalities including sprint, sprint interval training, and resistance exercise. The most widely reported improvements to performance with CM were outcomes related to muscular strength-endurance, explosive exercise ability, and repetition volume. Importantly, CM enhancement of anaerobic/resistance exercise performance appears to be most pronounced when applied during exercise and when repeated efforts are necessary with single efforts (e.g., maximal strength) showing comparatively less efficacy. Regarding psychophysiological outcomes, RPE, motivation, arousal, and affect were most commonly reported but showed more heterogenous responses. However, enhancement of psychophysiological outcomes were reported even in the absence of performance enhancement, suggesting CM may induce benefits important for coping or sustaining high-intensity work.

Of the 19 studies included that measured indicators of anaerobic/resistance performance, 16 studies (84%) reported enhancement of at least one performance-based outcome. This was most reflected in enhancement of power-based metrics and repetition volume. In anaerobic sprint and sprint interval modalities, CM was reported to enhance power output and cause faster sprint times. For example, Meglic et al. showed increases in mean power and total work during repeated Wingate sprints in female collegiate athletes with CM versus non-preferred music [48]. Further supporting this, Stork et al. showed CM-induced enhancement of peak and mean power during repeated Wingate anaerobic sprint tests versus no music [52]. Jebabli et al. also observed faster sprint times with CM versus no music in trained soccer athletes [45]. While it has been suggested that CM may impart benefits to endurance-based exercise performance through pacing, this is unlikely applicable to current findings of increased power output as maximal anaerobic test performance is less subject to pacing effects. Current findings of improved power-based outcomes are likely more related to changes in motivation and arousal. Indeed, music may impart a “psyching up” effect, thereby enhancing arousal, effort allocation, and power development [15,59]. Regarding resistance exercise, numerous investigations have reported improvements in explosive ability and repetition volume. Ballmann et al. has shown enhancement of repetition volume during bench press with CM administered pre-task, warm-up, and during repeated exercise [9,38,39]. Bolstering this further, multiple investigations have shown enhancement of force during isometric tests with increases in force production [15,42,51]. However, dynamic maximal strength appears to be less affected by CM. For example, Bartolomei et al. showed no changes in one-repetition maximum performance with CM versus no music [40]. From a mechanistic standpoint, muscular strength is heavily influenced by neuromuscular capacity and muscle size while repetition volume is more dependent on acute changes in neuromuscular drive and fatigue resistance. CM may be able to more readily modulate factors related to acute neural activation and fatigue than neuromuscular capacity, which may take more chronic training strategies to enhance. Furthermore, CM-induced dissociative or distracting effects may not be potent enough to beneficially affect dynamic strength. Indeed, evidence has suggested strong external focus during maximal effort exercise is beneficial to performance in both acute and chronic contexts [60]. This is largely speculative at this time, and future studies should specifically seek to understand how CM-induced dissociation may directly affect dynamic strength.

CM-induced alterations in psychophysiological outcomes were also widely reported in anaerobic/resistance exercise investigations even in the absence of performance enhancement. By far, the most commonly reported outcome was increased motivation with CM. Indeed, 10 studies included motivation as an outcome during anaerobic/resistance exercise, with 9 (90%) reporting positive effects. Ballmann et al. showed enhanced motivation during exercise when CM was applied during pre-task, warm-up, and throughout exercise during bench press in resistance-trained males [9,38,39]. Increases in motivation were also apparent in multiple studies investigating CM and anaerobic sprint performance [13,41]. While not confirmed, increases in motivation with CM are likely a result of increased engagement of reward pathways in the brain. Indeed, music results in greater dopamine release affecting reward pathway activation but responses are mediated by CM [18,61,62]. This may in turn result in increased goal-directed behavior and motivational drive, thus improving effort during exercise. These effects may also explain concomitant enhancement in arousal and affect. For example, Greco et al. showed improved affect with CM during isometric leg exercise while Rogers et al. reported increased motivation and arousal levels during isometric mid-thigh pull testing [15,44]. Taken together, CM appears to potently affect affective-motivational pathways, thereby enhancing effort allocation, increasing affective tolerance of intense exercise, and leading to hypermotivated states. Interestingly, recent evidence by Zhang et al. shows more direct evidence of changes in neural drive with CM [20]. Authors revealed that CM during a warm-up resulted in reduced cortical inhibition and enhanced readiness to give effort which was accompanied by improved sprint performance. When taken together with other reports of increased force development [15], barbell velocity [9,38], and neuromuscular excitation [42], it is likely that CM acutely enhances neural drive while modulating inhibitory feedback loops that may mediate maximal anaerobic performance. While intriguing, more investigations elucidating the mechanistic underpinnings of CM-induced ergogenic effects are warranted to support previous postulated mechanisms.

### 4.3. Psychophysiological Framework

**Figure 2:**
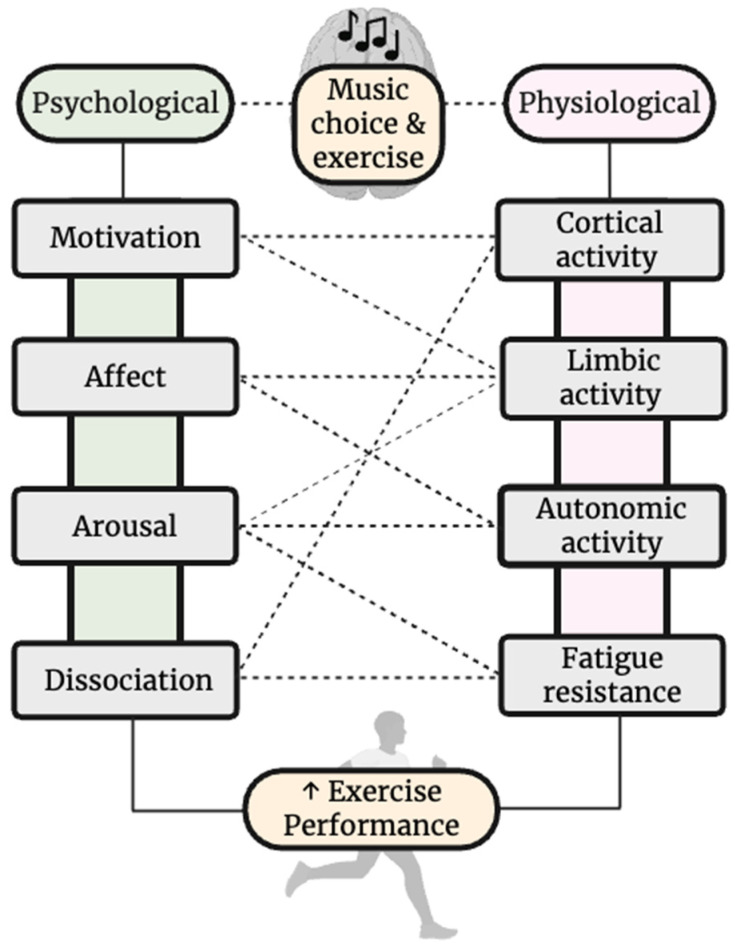
Framework matrix of purported psychophysiologic underpinnings of ergogenic benefits from choice music (CM) during exercise.

From the studies reviewed, a psychophysiological framework matrix (Figure 2) was developed to explain underpinning mechanisms responsible for CM-induced performance enhancement. CM induces changes spanning both psychological and physiological domains [2,63]. Listening to CM increases motivation, which has been previously linked to enhancement of brain activity in cortical and limbic regions, thereby enhancing reward, neural drive, and effort allocation [18,61,62]. CM also improves affect and feelings of enjoyment, likely through limbic and autonomic modulation, which together may improve emotional responses and willingness to give effort during exercise [8,11,52,64]. Arousal or feelings of being “psyched up” by CM have been suggested to be under the influences of limbic and autonomic activity, which may lead to improved ability to resist fatigue and give maximal effort [15,65]. Reported changes in RPE and dissociation may be a result of altered cortical activity and lead to enhanced fatigue resistance and ability to sustain high workloads [20,33,66].

### 4.4. Limitations

Despite the review providing strong evidence for the use of CM as an effective ergogenic aid across a host of exercise modalities, numerous limitations and challenges exist. Heterogeneity in study design, exercise modalities, participant characteristics, and operational definitions of “self-selected” or “preference” for music interventions greatly constrain conclusions due to difficulty in making direct comparisons across studies. In particular, included studies were largely focused on male participants rather than females. Since previous evidence has suggested that psychophysiological responses to CM in females may be more favorable than males [50], future studies will need to include both sexes or directly compare responses in order to make widespread recommendations of CM for practical implementation. Furthermore, many of the neural mechanisms discussed remain speculative as well controlled experiments specifically addressing mechanistic underpinnings of CM-induced ergogenic benefits are sparse. Lastly, there is little to no evidence available that has accounted for variations in music characteristics (e.g., tempo, lyrics, timbre) in the context of music preference, despite likely contributions to differences in responses. Future research should incorporate standardized protocols and reporting, establish neuromechanistic rationale for ergogenic benefit, and identify how choice and preference for specific attributes of music potentially alter exercise responses.

## 5. Conclusions

Synthesis of the reviewed literature strongly supports the efficacy of CM compared to non-preferred or pre-determined music, which reinforces the need for individualized music interventions to promote a high degree of efficacy. Both endurance and anaerobic/resistance exercise performance were widely reported to be impacted, which were largely mediated by distinct psychophysiological benefits including alterations in perceived exertion, motivation, affect, and neural drive.

## 6. Practical Applications

From a practical standpoint, the current review strongly suggests that CM may be a valuable adjunctive training tool for athletes and recreational exercisers alike. Personalizing music interventions for athletes may enhance the ergogenic potential of music while promoting autonomy and motivation to train. Importantly, CM represents a feasible, low-cost, and highly accessible means to improve psychophysiological processes which mediate exercise performance. Athletes and exercisers may strategically leverage CM by curating readily available music playlists for particular modes or intensities of exercise training. Further refinement of mechanisms to translate into practice is necessary to understand how CM can be strategically leveraged to optimize human performance and exercise training.

## Figures and Tables

**Table 1:** Reviewed studies on psychophysiological responses and performance during aerobic/endurance exercise (n = 13).

Study	Participants (N, Sex, Age)	Training/Health Status	Music Condition(s)	Exercise Task/Modality	Effect(s) of CM
Clark et al. 2021 [28]	N = 17 (8 M/9 F), Mean age: 24.2 ± 4.9	Recreational runners	SSM vs. No Music	1.5-mile run time trial	↔ Time to completion, ↔ Heart rate, ↓ RPE
Cole et al. 2015 [30]	N = 35 (15 M/20 F), Mean age: 20.7 ± 2.3	Recreational runners	Pref vs. No Music	Cooper (12 min) run	↑ Distance (Females only)
Filho et al. 2022 [31]	N = 11 (11 M/0 F), Mean age: 26.4 ± 2.1	Physically active	Pref vs. Nonpref vs. No Music	High-Intensity Interval Training with Bodyweight	↑ Repetitions, ↑ affective response, ↔ RPE, ↔ heart rate, ↔ mood state
Hagen et al. 2013 [32]	N = 18 (9 M/9 F), Age: Not Reported	Trained cyclists	SSM vs. No music	10 km cycling time trial	↔ Time to completion, ↔ mean power, ↔ heart rate, ↔ blood lactate, ↔ RPE.
Hutchinson et al. 2018 [33]	N = 17 (9 M/8 F), Mean age: 28.1 ± 9.9	Physically active	SSM vs. No music	Treadmill exercise	↑ Exercise intensity, ↑ feeling scale, ↑ pleasure
Jebabli et al. 2020 [26]	N = 20 (20 M/0 F), Mean age: 22 ± 1.3	Physically active	Pref vs. No music	6 min run test	↑ Mean running speed, ↑ total distance, ↔ RPE, ↔ heart rate, ↔ blood lactate
Jebabli et al. 2022 [34]	N = 25 (25 M/0 F), Mean age: 21.0 ± 1.1	Middle-distance runners	Pref vs. No music	6 min run test	↑ Total distance, ↔ heart rate, ↓ blood lactate, ↔ affect, ↔ RPE
Karow et al. 2021 [29]	N = 12 (6 M/6 F), Mean age = 21.1 ± 1.0	Physically active	Pref vs. Nonpref vs. No music (warm-up)	2000 m rowing time trial	↑ Relative power output,↓ Time to completion, ↑ Heart rate, ↔ RPE, ↑ Motivation
Labudovic et al. 2024 [27]	N = 20 (0 M/20 F), Age: College-aged	Sport and Physical Education majors	Synchronized SSM vs. no music	Cardiopulmonary Exercise Test (Aerobic)	↑ Exercise duration, ↑ peak VO_2_, ↑ peak heart rate, ↓ RPE
Lane et al. 2011 [35]	N = 60 (19 M/41 F), Mean age: 41.48 ± 9.39	Recreational and competitive runners	SSM vs. commercial playlist	Running goal attainment (Aerobic)	↑ Self-reported performance, ↑ emotional state
Nakamura et al. 2010 [7]	N = 15 (15 M/0 F), Mean age: 22.8 ± 3.1	Recreationally active cyclists	Pref vs. Nonpref	Critical power test (cycling)	↑ Distance, ↔ heart rate, ↓ RPE
Rasterio et al. 2020 [36]	N = 20 (10 M/10 F), Mean age: 23–24	Physically active	Pref vs. No music	Incremental running test to exhaustion	↑ Time to exhaustion, ↑ speed at anaerobic threshold, ↑ blood lactate, ↑ heart rate, ↑ RPE (Females only)
Tanaka et al. 2018 [37]	N = 15 (15 M/0 F), Mean age: 22.9 ± 0.5	Physically active	SSM vs. No music	Cycling (20 min)	↑ Cognitive task performance, ↔ arousal, ↓ RPE

Increases (↑), decreases (↓), and no change (↔) are noted for all relevant outcomes. M = male, F = female, SSM = self-selected music, Pref = Preferred music, Nonpref = non-preferred music, RPE = rating of perceived exertion, VO_2_ = oxygen consumption.

**Table 2:** Reviewed studies on psychophysiological responses and performance during anaerobic/resistance exercise (n = 19).

Study	Participants (N, Sex, Age)	Training/Health Status	Music Condition(s)	Exercise Task/Modality	Effect(s) of CM
Ballmann et al. 2021 [9]	N = 12 (12 M/0 F), Mean age: 20.5 ± 1.24	Resistance Trained	Pref vs. Nonpref	Bench Press	↑ Reps to failure, ↑ velocity, ↑ power, ↑ motivation
Ballmann et al. 2021 [38]	N = 10 (10 M/0 F), Mean age: 22.8 ± 5.8	Resistance Trained	SSM vs. No music (Pre-task)	Bench Press	↑ Reps to failure, ↑ velocity, ↑ power, ↑ motivation
Ballmann et al. 2020 [39]	N = 10 (10 M/0 F), Mean = 21.6 ± 1.7	Resistance Trained	Pref vs. Nonpref (Warm-up)	Bench Press	↑ Reps to failure, ↔ velocity, ↑ motivation, ↔ RPE
Ballmann et al. 2019 [13]	N = 14 (14 M/0 F), Mean = 20.1 ± 1.7	Physically active	Pref vs. Nonpref	Wingate Anaerobic Test (Repeated)	↔ Anaerobic power, ↔ Anaerobic capacity, ↔ Total work, ↔ Heart rate, ↑ motivation, ↓ RPE
Bartolomei et al. 2015 [40]	N = 31 (31 M/0 F), Age: 21.6–26.6	Resistance-trained men	SSM vs. No music	Bench Press	↔ Maximal strength, ↑ Repetition volume
Cavaggioni et al. 2025 [41]	N = 18 (12 M/6 F), Mean age: 22.2 ± 2.1	Sub-élite athletes	Pref vs. No music	Anaerobic sprint	↔ Power output, ↔ RPE, ↑ motivation
Cotellessa et al. 2024 [42]	N = 28 (18 M/10 F), Mean age: 29.57 ± 2.77	Untrained and trained individuals	SSM vs. No music	Isometric bicep flexion to exhaustion	↑ Task duration, ↑ muscle activation (Untrained only)
Cutrufello et al. 2019 [43]	N = 15 (8 M/7 F), Mean age: 20.1 ± 1.7	Healthy college-aged	SSM vs. No music	Wingate test and Bench Press	↑ Repetitions to failure, ↑ total work, ↑ power output, ↑ heart rate recovery
Greco et al. 2022 [44]	N = 26 (26 M/0 F), Mean age: 50.8 ± 8.4	Healthy physically active	SSM vs. No music	Isometric leg extension	↑ Force, ↔ RFD ↔ fatigue index, ↑ arousal, ↔ RPE, ↑ affect
Jebabli et al. 2023 [45]	N = 19 (19 M/0 F), Mean age: 22.1 ± 1.2	Semi-professional soccer players	Pref vs. No music	20 m sprints (Repeated)	↓ Total sprint time, ↔ heart rate, ↑ blood lactate, ↔ affect, ↔ RPE
Lehman et al. 2021 [46]	N = 10 (10 M/0 F), Mean age: 20.7 ± 1.1	Resistance trained	SSM vs. No music (Respite)	Bench press	↔ Reps to failure, ↑ velocity, ↑ power, ↑ motivation, ↔ RPE
Marques et al. 2022 [47]	N = 16 (16 M/0 F), Mean age: 27.0 ± 3.9	Physically active	Self-selected vs. experimenter-selected music vs. no music	Sprint interval training (cycling)	↔ Power output, ↔ fatigue index, ↔ total work, ↔ RPE, ↔ enjoyment, ↓ attentional focus
Meglic et al. 2021 [48]	N = 14 (0 M/14 F), Mean age: 19.9 ± 1.3	Division 1 NCAA Athletes	Pref vs. Nonpref (Warm-up)	Wingate Anaerobic Test (repeated)	↑ Mean power, ↑ total work, ↔ RPE, ↑ motivation
Moss et al. 2018 [49]	N = 16 (16 M/0 F), Mean age: 22 ± 3.4	Resistance trained	SSM vs. No music	Back squat and bench press throw	↔ Mean power, ↔ velocity, ↑ reps to failure, ↔ mood, ↓ RPE
Rhoads et al. 2021 [50]	N = 16 (8 M/8 F), Mean age: 21.6 ± 1.7	Physically active	SSM vs. No music	Wingate Anaerobic Test (repeated)	↔ Relative power, ↓ fatigue index, ↓ RPE, ↑ motivation (Females only)
Rogers et al. 2023 [15]	N = 12 (12 M/0 F), Mean age: 20.9 ± 0.3	Physically active	Pref vs. Nonpref	Isometric mid-thigh pull and countermovement jump	↑ Peak force, ↑ RFD, ↔ jump height, ↔ peak power, ↑ motivation, ↑ arousal.
Silva et al. 2021 [51]	N = 20 (20 M/0 F), Mean age: 20.0 ± 1.4	Resistance trained	Pref vs. Nonpref vs. No music	Grip strength, Lat pulldown	↑ Grip Strength, ↑ Reps to failure, ↓ RPE
Stork et al. 2015 [52]	N = 20 (10 M/10 F), Mean age: 22.5 ± 4.3	Moderately active	SSM vs. No music	Wingate Anaerobic Test (repeated)	↑ Peak power, ↑ mean power, ↔ RPE, ↔ motivation, ↑ enjoyment
Zhang et al. 2023 [20]	N = 17 (17 M/0 F), Mean age: 21.3 ± 1.0	Healthy young men	SSM vs. No music (Warm-up)	Wingate Anaerobic Test	↑ Power output, ↔ fatigue index, ↑ Cortical activity (EEG), ↔ heart rate, ↔ RPE

Increases (↑), decreases (↓), and no change (↔) are noted for all relevant outcomes. M = male, F = female, SSM = self-selected music, Pref = Preferred music, Nonpref = non-preferred music, RPE = rating of perceived exertion, Reps = repetitions, RFD = rate of force development.

## Data Availability

No new data were created or analyzed in this study. Data sharing is not applicable to this article.

## Supplementary Materials

The following supporting information can be downloaded at: https://www.mdpi.com/article/10.3390/jfmk11020144/s1. Search string terms for each database are detailed in File S1.

## References

[1] Bannan N., Harvey A.R. (2025). Music as a social instrument: A brief historical and conceptual perspective. Front. Cogn..

[2] Ballmann C.G. (2021). The influence of music preference on exercise responses and performance: A review. J. Funct. Morphol. Kinesiol..

[3] Grgic J. (2022). Effects of music on resistance exercise performance: A narrative review. Strength Cond. J..

[4] Schwartz S.E., Fernhall B., Plowman S.A. (1990). Effects of music on exercise performance. J. Cardiopulm. Rehabil. Prev..

[5] Karageorghis C.I., Priest D.-L. (2012). Music in the exercise domain: A review and synthesis (Part I). Int. Rev. Sport Exerc. Psychol..

[6] Franco-Alvarenga P.E., Brieztke C., Canestri R., Pires F.O. (2019). Psychophysiological responses of music on physical performance: A critical review. Rev. Bras. Ciência Mov..

[7] Nakamura P.M., Pereira G., Papini C.B., Nakamura F.Y., Kokubun E. (2010). Effects of preferred and nonpreferred music on continuous cycling exercise performance. Percept. Mot. Ski..

[8] Nixon K.M., Parker M.G., Elwell C.C., Pemberton A.L., Rogers R.R., Ballmann C.G. (2022). Effects of music volume preference on endurance exercise performance. J. Funct. Morphol. Kinesiol..

[9] Ballmann C.G., McCullum M.J., Rogers R.R., Marshall M.R., Williams T.D. (2021). Effects of preferred vs. nonpreferred music on resistance exercise performance. J. Strength Cond. Res..

[10] Juslin P.N., Västfjäll D. (2008). Emotional responses to music: The need to consider underlying mechanisms. Behav. Brain Sci..

[11] Terry P.C., Karageorghis C.I., Curran M.L., Martin O.V., Parsons-Smith R.L. (2020). Effects of music in exercise and sport: A meta-analytic review. Psychol. Bull..

[12] Bigliassi M., Estanislau C., Carneiro J.G., Kanthack T.F.D., Altimari L.R. (2013). Music: A psychophysiological aid to physical exercise and sport. Arch. Med. Deporte.

[13] Ballmann C.G., Maynard D.J., Lafoon Z.N., Marshall M.R., Williams T.D., Rogers R.R. (2019). Effects of listening to preferred versus non-preferred music on repeated wingate anaerobic test performance. Sports.

[14] Potteiger J.A., Schroeder J.M., Goff K.L. (2000). Influence of music on ratings of perceived exertion during 20 minutes of moderate intensity exercise. Percept. Mot. Ski..

[15] Rogers R.R., Williams T.D., Nester E.B., Owens G.M., Ballmann C.G. (2023). The Influence of Music Preference on Countermovement Jump and Maximal Isometric Performance in Active Females. J. Funct. Morphol. Kinesiol..

[16] Salimpoor V.N., Benovoy M., Larcher K., Dagher A., Zatorre R.J. (2011). Anatomically distinct dopamine release during anticipation and experience of peak emotion to music. Nat. Neurosci..

[17] Vuust P., Heggli O.A., Friston K.J., Kringelbach M.L. (2022). Music in the brain. Nat. Rev. Neurosci..

[18] Salimpoor V.N., Van Den Bosch I., Kovacevic N., McIntosh A.R., Dagher A., Zatorre R.J. (2013). Interactions between the nucleus accumbens and auditory cortices predict music reward value. Science.

[19] Cook T., Roy A.R., Welker K.M. (2019). Music as an emotion regulation strategy: An examination of genres of music and their roles in emotion regulation. Psychol. Music.

[20] Zhang S., Yang J., Tao X., Du L., Li X., Lv Y., Hou X., Yu L. (2023). Listening to self-selected music during warm-up improves anaerobic performance through enhancement of the excitability of the cerebral cortex. Appl. Sci..

[21] Yamamoto T., Ohkuwa T., Itoh H., Kitoh M., Terasawa J., Tsuda T., Kitagawa S., Sato Y. (2003). Effects of pre-exercise listening to slow and fast rhythm music on supramaximal cycle performance and selected metabolic variables. Arch. Physiol. Biochem..

[22] Yamashita S., Iwai K., Akimoto T., Sugawara J., Kono I. (2006). Effects of music during exercise on RPE, heart rate and the autonomic nervous system. J. Sports Med. Phys. Fit..

[23] Page M.J., McKenzie J.E., Bossuyt P.M., Boutron I., Hoffmann T.C., Mulrow C.D., Shamseer L., Tetzlaff J.M., Akl E.A., Brennan S.E. (2021). The PRISMA 2020 statement: An updated guideline for reporting systematic reviews. BMJ.

[24] Tricco A.C., Lillie E., Zarin W., O’Brien K.K., Colquhoun H., Levac D., Moher D., Peters M.D., Horsley T., Weeks L. (2018). PRISMA extension for scoping reviews (PRISMA-ScR): Checklist and explanation. Ann. Intern. Med..

[25] Liguori G. (2020). ACSM’s Guidelines for Exercise Testing and Prescription.

[26] Jebabli N., Granacher U., Selmi M.A., Al-Haddabi B., Behm D.G., Chaouachi A., Haj Sassi R. (2020). Listening to preferred music improved running performance without changing the pacing pattern during a 6 minute run test with young male adults. Sports.

[27] Labudović D., Stojiljković S., Orlić A., Matić M., Uzunović S., Bubanj S., Dobrescu T., Macura M., Popović D. (2024). Impact of Music Selection on Motivation and Performance during Cardiopulmonary Exercise Testing. Appl. Sci..

[28] Clark J.C., Baghurst T., Redus B.S. (2021). Self-selected motivational music on the performance and perceived exertion of runners. J. Strength Cond. Res..

[29] Karow M.C., Rogers R.R., Pederson J.A., Williams T.D., Marshall M.R., Ballmann C.G. (2020). Effects of preferred and nonpreferred warm-up music on exercise performance. Percept. Mot. Ski..

[30] Cole Z., Maeda H. (2015). Effects of listening to preferential music on sex differences in endurance running performance. Percept. Mot. Ski..

[31] Allocca Filho R.A., Oliveira J.J., Zovico P.V.C., Rica R.L., Barbosa W.A., Machado A.F., Evangelista A.L., Costa E.C., Bergamin M., Baker J.S. (2022). Effects of music on psychophysiological responses during high intensity interval training using body weight exercises. Physiol. Behav..

[32] Hagen J., Foster C., Rodríguez-Marroyo J., De Koning J.J., Mikat R.P., Hendrix C.R., Porcari J.P. (2013). The effect of music on 10-km cycle time-trial performance. Int. J. Sports Physiol. Perform..

[33] Hutchinson J.C., Sherman T. (2014). The relationship between exercise intensity and preferred music intensity. Sport Exerc. Perform. Psychol..

[34] Jebabli N., Zouhal H., Boullosa D., Govindasamy K., Tourny C., Hackney A.C., Granacher U., Abderrahman A.B. (2022). The effects of preferred music and its timing on performance, pacing, and psychophysiological responses during the 6-min test. J. Hum. Kinet..

[35] Lane A.M., Davis P.A., Devonport T.J. (2011). Effects of music interventions on emotional states and running performance. J. Sports Sci. Med..

[36] Rasteiro F.M., Messias L.H.D., Scariot P.P.M., Cruz J.P., Cetein R.L., Gobatto C.A., Manchado-Gobatto F.B. (2020). Effects of preferred music on physiological responses, perceived exertion, and anaerobic threshold determination in an incremental running test on both sexes. PLoS ONE.

[37] Tanaka D., Tsukamoto H., Suga T., Takenaka S., Hamaoka T., Hashimoto T., Isaka T. (2018). Self-selected music-induced reduction of perceived exertion during moderate-intensity exercise does not interfere with post-exercise improvements in inhibitory control. Physiol. Behav..

[38] Ballmann C.G., Favre M.L., Phillips M.T., Rogers R.R., Pederson J.A., Williams T.D. (2021). Effect of pre-exercise music on bench press power, velocity, and repetition volume. Percept. Mot. Ski..

[39] Ballmann C.G., Cook G.D., Hester Z.T., Kopec T.J., Williams T.D., Rogers R.R. (2020). Effects of preferred and non-preferred warm-up music on resistance exercise performance. J. Funct. Morphol. Kinesiol..

[40] Bartolomei S., Michele R.D., Merni F. (2015). Effects of self-selected music on maximal bench press strength and strength endurance. Percept. Mot. Ski..

[41] Cavaggioni L., Formenti D., Ouergui I., Perpetuini D., Castiglioni P., Berengan A., Trecroci A., Ardigo L.P., Merati G. (2025). Effects of music listening on anaerobic performance and motivation in healthy young adults. Front. Sports Act. Living.

[42] Cotellessa F., Bragazzi N.L., Trompetto C., Marinelli L., Mori L., Faelli E., Schenone C., Ceylan H.İ., Biz C., Ruggieri P. (2024). Improvement of motor task performance: Effects of verbal encouragement and music—Key results from a randomized crossover study with electromyographic data. Sports.

[43] Cutrufello P.T., Benson B.A., Landram M.J. (2019). The effect of music on anaerobic exercise performance and muscular endurance. J. Sports Med. Phys. Fit..

[44] Greco F., Rotundo L., Grazioli E., Parisi A., Carraro A., Muscoli C., Paoli A., Marcolin G., Emerenziani G.P. (2022). Effects of self-selected versus motivational music on lower limb muscle strength and affective state in middle-aged adults. PeerJ.

[45] Jebabli N., Ben Aabderrahman A., Boullosa D., Chtourou H., Ouerghi N., Rhibi F., Govindasamy K., Saeidi A., Clark C.C., Granacher U. (2023). Listening to music during a repeated sprint test improves performance and psychophysiological responses in healthy and physically active male adults. BMC Sports Sci. Med. Rehabil..

[46] Lehman J.T., Whitmire B.G., Rogers R.R., Williams T.D., Ballmann C.G. (2021). Effects of respite music on repeated upper-body resistance exercise performance. Int. J. Exerc. Sci..

[47] Marques M., Staibano V., Franchini E. (2022). Effects of self-selected or randomly selected music on performance and psychological responses during a sprint interval training session. Sci. Sports.

[48] Meglic C.E., Orman C.M., Rogers R.R., Williams T.D., Ballmann C.G. (2021). Influence of warm-up music preference on anaerobic exercise performance in division I NCAA female athletes. J. Funct. Morphol. Kinesiol..

[49] Moss S.L., Enright K., Cushman S. (2018). The influence of music genre on explosive power, repetitions to failure and mood responses during resistance exercise. Psychol. Sport Exerc..

[50] Rhoads K.J., Sosa S.R., Rogers R.R., Kopec T.J., Ballmann C.G. (2021). Sex Differences in Response to Listening to Self-Selected Music during Repeated High-Intensity Sprint Exercise. Sexes.

[51] Silva N.R.d.S., Rizardi F.G., Fujita R.A., Villalba M.M., Gomes M.M. (2021). Preferred music genre benefits during strength tests: Increased maximal strength and strength-endurance and reduced perceived exertion. Percept. Mot. Ski..

[52] Stork M.J., Kwan M.Y., Gibala M.J., Martin Ginis K.A. (2015). Music enhances performance and perceived enjoyment of sprint interval exercise. Med. Sci. Sports Exerc..

[53] Michaelis K., Wiener M., Thompson J.C. (2014). Passive listening to preferred motor tempo modulates corticospinal excitability. Front. Hum. Neurosci..

[54] Wilkins R.W., Hodges D.A., Laurienti P.J., Steen M., Burdette J.H. (2014). Network science and the effects of music preference on functional brain connectivity: From Beethoven to Eminem. Sci. Rep..

[55] Nozaradan S. (2014). Exploring how musical rhythm entrains brain activity with electroencephalogram frequency-tagging. Philos. Trans. R. Soc. B Biol. Sci..

[56] Brownley K.A., McMurray R.G., Hackney A.C. (1995). Effects of music on physiological and affective responses to graded treadmill exercise in trained and untrained runners. Int. J. Psychophysiol..

[57] Osuch E.A., Bluhm R.L., Williamson P.C., Théberge J., Densmore M., Neufeld R.W. (2009). Brain activation to favorite music in healthy controls and depressed patients. NeuroReport.

[58] Bigliassi M., Karageorghis C.I., Bishop D.T., Nowicky A.V., Wright M.J. (2018). Cerebral effects of music during isometric exercise: An fMRI study. Int. J. Psychophysiol..

[59] Ballmann C.G., Porrill S.L., Rogers R.R., Ervin Z.H., Neal B.R., Nguyen H.M., Spears P.N., Strickland J.E., Zavala J., Washmuth N.B. (2025). Effects of Censoring Explicit Language in Music on Resistance Exercise Performance. J. Funct. Morphol. Kinesiol..

[60] Grgic J., Mikulic I., Mikulic P. (2021). Acute and long-term effects of attentional focus strategies on muscular strength: A meta-analysis. Sports.

[61] Blum K., Chen T.J., Chen A.L., Madigan M., Downs B.W., Waite R.L., Braverman E.R., Kerner M., Bowirrat A., Giordano J. (2010). Do dopaminergic gene polymorphisms affect mesolimbic reward activation of music listening response? Therapeutic impact on Reward Deficiency Syndrome (RDS). Med. Hypotheses.

[62] Brodal H.P., Osnes B., Specht K. (2017). Listening to rhythmic music reduces connectivity within the basal ganglia and the reward system. Front. Neurosci..

[63] Karageorghis C.I., Terry P.C. (1997). The psychophysical effects of music in sport and exercise: A review. J. Sport Behav..

[64] Urakawa K., Yokoyama K. (2005). Music can enhance exercise-induced sympathetic dominancy assessed by heart rate variability. Tohoku J. Exp. Med..

[65] Hove M.J., Martinez S.A., Shorrock S.R. (2022). Physical exercise increases perceived musical pleasure: Modulatory roles of arousal, affect, or dopamine?. Psychol. Music.

[66] Szmedra L., Bacharach D. (1998). Effect of music on perceived exertion, plasma lactate, norepinephrine and cardiovascular hemodynamics during treadmill running. Int. J. Sports Med..

