# Supplementary material for: Effects of Music Choice on Performance and Psychophysiological Responses to Exercise—A Scoping Review
Source: J Funct Morphol Kinesiol. 2026 Mar 31;11(2):144. doi: 10.3390/jfmk11020144 (PMC13108026; doi:10.3390/jfmk11020144)
Supplement: Supplementary file 1 [file jfmk-11-00144-s001.zip › jfmk-4196357-supplementary.pdf]

Electronic Supplementary Material File S1

Bibliographic Literature searching

Database	Hits
CINAHL Plus with Full Text	160
Embase	1189
PubMed	1381
Scopus	702
Web of Science	1504
Total	4936
- duplicates	1078
Unique studies to screen	3858

Database: CINAHL Plus with Full Text
Host: EBSCOhost
Data Parameters: 1937 to Present
Date Searched: April 30, 2025
Searcher: Rebecca Billings

Search Strategy:

#	Searches	Results
1	(MM "Music Therapy" AND MM "Decision Making") OR ((music OR "binaural beats" OR genre* OR playlist* OR rhythm* OR song OR songs OR sound* OR tempo) N3 (choice* OR decision* OR favorite* OR favourite* OR individual* OR individualized OR self-select* OR select* OR personal* OR prefer* OR non-prefer* OR predetermined)))	2710
2	(TI alertness OR alpha-wave* OR "alpha rhythm" OR TI anxiet* OR TI arousal OR MM "Attention" OR TI attention OR "autonomic nervous system" OR "autonomic innervation" OR "adrenergic transmission" OR beta-wave* OR "beta rhythm" OR "brain activity" OR MM "Brain Waves+" OR brain-wave* OR "cardio vagal" OR "cognitive control" OR MM "Cognition" OR cognitive-function* OR TI concentrat* OR delta-wave* OR "delta rhythm" OR MM "Electrocardiography" OR electrocardiograph* OR MM "Electroencephalography" OR electroencephalograph* OR MM "Executive Function" OR executive-function* OR TI exercise OR TI fitness OR TI focus OR MM "Heart Rate" OR MM "Heart Rate Variability" OR "heart rate" OR MM "Psychomotor Performance" OR MM "Physical Performance" OR MM "Psychomotor Disorders" OR MM "Mental Fatigue" OR "mental fatigue" OR MM "Motor Skills" OR MM "Motor Activity" OR motor-abilit* OR motor-function* OR motor-skill* OR motor-performance* OR non-motor-	967245

	function* OR TI "nervous system" OR "neural activation" OR neural-respons* OR parasympathetic OR psychomotor-performance* OR MM "Stress, Psychological" OR MM "Stress" OR MM "Stress, Physiological" OR stress* OR MM "Task Performance and Analysis" OR MM "Physical Performance" OR performance OR "physical exertion" OR "psychophysiological outcomes" OR MM "Reaction Time" OR reaction-time* OR "reaction latency" OR "response time" OR "response latency" OR TI regulat* OR "stimulus response" OR TI sympathetic OR "theta waves" OR "theta rhythm" OR MM "Vagus Nerve" OR "vagus nerve")	
3	(MH "Clinical Trials" OR MH "Comparative Studies" OR MH "Randomized Controlled Trials" OR MH "Nonrandomized Trials" OR MH "Control Group" OR MH "Double-Blind Studies" OR MH "Empirical Research" OR MH "Single-Blind Studies" OR MH "Epidemiological Research" OR MH "Factorial Design" OR MH "Quasi-Experimental Studies" OR MH "Nonequivalent Control Group" OR clinical-stud* OR pretest* OR posttest* OR quasi-experiment* OR "parallel design" OR intervention*)	1372653
4	#1 AND #2 AND #3	426
5	#4 NOT (MH "Literature Review" OR MH "Systematic Review" OR MH "Scoping Review" OR TI review* OR MH "Chronic Disease+" OR MH "Noncommunicable Diseases" OR chronic-disease* OR chronic-illness* OR chronically-ill* OR noncommunicable-disease* OR musician* OR musician* OR performing-artist*)	389
6	Filters: Abstract available; Human; English; 2000-present	160

Database: Embase
Host: Elsevier
Data Parameters: 1947 to Present
Date Searched: April 30, 2025
Searcher: Rebecca Billings

Search Strategy:

#	Searches	Results
1	('music'/mj/exp OR 'music therapy'/exp OR music:ab,ti) AND ('attention'/exp OR 'autonomic nervous system'/exp OR 'autonomic nervous system function'/exp OR 'autonomic innervation':ab,ti OR 'adrenergic transmission':ab,ti OR 'parasympathetic tone':ab,ti OR 'cognitive function*':ab,ti OR 'electrocardiograph'/exp OR electrocardiograph*:ab,ti OR 'electroencephalogram'/exp/mj OR 'executive function'/exp OR 'executive function*':ab,ti OR 'heart'/mj OR 'heart rate'/exp OR 'heart rate':ab,ti OR 'motor performance'/exp OR 'mental stress'/exp OR 'neural activation':ab,ti OR stress*:ab,ti OR 'performance'/exp OR performance:ab,ti OR 'physiological stress'/exp/mj OR 'physiology'/mj OR 'psychology'/mj	4969

	OR 'psychophysiological outcomes':ab,ti OR 'reaction time'/exp OR 'reaction time*':ab,ti OR 'vagus nerve'/exp OR 'vagus nerve':ab,ti) AND ('decision making'/de OR decision* OR choice*:ab,ti,kw OR favorite:ab,ti,kw OR favourite:ab,ti,kw OR individual*:ab,ti,kw OR 'self select*':ab,ti,kw OR select*:ab,ti,kw OR personal*:ab,ti,kw OR prefer*:ab,ti,kw OR 'non prefer*':ab,ti,kw OR predetermined:ab,ti,kw)	
2	((choice* OR favorite OR favourite OR individual* OR 'self select*' OR select* OR personal* OR prefer* OR 'non prefer*' OR predetermined) NEAR/6 ('binaural beats' OR genre OR listening OR music* OR playlist* OR rhythm* OR song OR songs OR sound* OR tempo) NEAR/6 (alertness OR anxiet* OR arousal OR attention* OR 'alpha wave*' OR 'alpha rhythm' OR 'autonomic nervous system' OR 'beta wave*' OR 'beta rhythm' OR 'brain activity' OR 'brain wave*' OR 'cardio vagal' OR 'cognitive control' OR concentrat* OR 'delta wave*' OR 'delta rhythm' OR eeg OR electrocardiograph* OR 'executive function' OR exercise OR fitness OR focus OR 'heart rate' OR 'motor abilit*' OR 'motor function*' OR 'motor performance*' OR 'motor skill*' OR 'non-motor function*' OR 'nervous system' OR 'neural activation' OR 'neural respons*' OR parasympathetic OR perform* OR 'physical exertion' OR physiology OR 'psychological arousal' OR 'psychophysiological outcome*' OR 'reaction time' OR 'reaction latency' OR 'response time' OR 'response latency' OR regulat* OR 'stimulus response' OR stress* OR sympathetic OR 'theta waves' OR 'theta rhythm' OR 'vagus nerve')):ab,ti,kw	1686
3	#1 OR #2 NOT ('performing artist'/exp OR 'musician'/exp OR musician*:ab,ti,kw OR 'performing artist*':ab,ti,kw) AND [2000-2025]/py	5260
4	#3 AND [adult]/lim AND [humans]/lim AND [english]/lim AND [abstracts]/lim	2462
5	#4 AND ('article'/it OR 'article in press'/it)	1777
6	#5 AND ('clinical study'/de OR 'clinical trial'/de OR 'comparative study'/de OR 'controlled clinical trial'/de OR 'controlled study'/de OR 'crossover procedure'/de OR 'double blind procedure'/de OR 'experimental design'/de OR 'experimental study'/de OR 'factorial design'/de OR 'human experiment'/de OR 'intervention study'/de OR 'major clinical study'/de OR 'parallel design'/de OR 'pretest posttest control group design'/de OR 'pretest posttest design'/de OR 'quasi experimental study'/de OR 'randomized controlled trial'/de OR 'single blind procedure'/de)	1189

Database: PubMed
Host: National Library of Medicine (http://www.ncbi.nlm.nih.gov/pubmed)
Data Parameters: 1946 to Present
Date Searched: April 30, 2025

Searcher: Rebecca Billings

Search Strategy:

#	Searches	Results
1	("Music Therapy"[Majr] OR "Music/psychology"[Majr] OR music[tiab] OR "binaural beats"[tiab] OR genre*[tiab] OR listening[ti] OR playlist*[tiab] OR rhythm*[tiab] OR song[tiab] OR songs[tiab] OR sound*[tiab] OR tempo[tiab]) AND ("Decision Making"[Majr] OR "Patient Preference"[Majr] OR choice*[tiab] OR decision*[tiab] OR favorite*[tiab] OR favourite*[tiab] OR individual*[ti] OR individualized[tiab] OR self-select*[tiab] OR select*[tiab] OR personal*[tiab] OR prefer*[tiab] OR non-prefer*[tiab] OR predetermined[tiab])	75370
2	(alertness[ti] OR "alpha wave*"[tiab] OR "Alpha Rhythm"[Majr] OR "alpha rhythm"[tiab] OR anxiet*[ti] OR arousal[ti] OR "Attention/physiology"[Majr] OR attention[ti] OR "Autonomic Nervous System"[Majr] OR "autonomic nervous system"[tiab] OR "autonomic innervation"[tiab] OR "adrenergic transmission"[tiab] OR beta-wave*[tiab] OR "Beta Rhythm"[Majr] OR "beta rhythm"[tiab] OR "brain activity"[tiab] OR "Brain Waves"[Majr] OR "brain wave*"[tiab] OR "cardio vagal"[tiab] OR "cognitive control"[tiab] OR "Cognition/physiology"[Majr] OR cognitive-function*[tiab] OR concentrat*[ti] OR delta-wave*[tiab] OR "Delta Rhythm"[Majr] OR "delta rhythm"[tiab] OR "Electrocardiography"[Majr] OR electrocardiograph*[tiab] OR "Electroencephalography"[Majr] OR electroencephalograph*[tiab] OR "Executive Function/physiology"[Majr] OR executive-function*[tiab] OR "Exercise/physiology"[Majr:NoExp] OR "Exercise/psychology"[Majr:NoExp] OR exercise[ti] OR fitness[ti] OR focus[ti] OR "Heart Rate"[Majr] OR "heart rate"[tiab] OR "Physical Fitness"[Majr] OR "Psychomotor Performance"[Majr] OR "Psychomotor Disorders"[Majr] OR "Mental Fatigue"[Majr] OR "mental fatigue"[tiab] OR "Motor Skills"[Majr] OR "Motor Activity"[Majr] OR motor-abilit*[tiab] OR motor-function*[tiab] OR motor-skill*[tiab] OR motor-performance*[tiab] OR non-motor-function*[tiab] OR "nervous system"[ti] OR "Neurofeedback"[Majr] OR "neural activation"[tiab] OR neural-respons*[tiab] OR parasympathetic[tiab] OR psychomotor-performance*[tiab] OR "Stress, Psychological"[Majr] OR stress*[tiab] OR "Performance Anxiety"[Majr] OR "Task Performance and Analysis"[Majr] OR "Physical Functional Performance"[Majr] OR performance[tiab] OR "Physiology"[Majr:NoExp] OR "physical exertion"[tiab] OR physiology[ti] OR "Psychophysiologic Disorders"[Majr] OR "psychophysiological outcomes"[tiab] OR "Reaction Time"[Majr] OR reaction-time*[tiab] OR "reaction latency"[tiab] OR "response time"[tiab] OR "response latency"[tiab] OR regulat*[ti] OR "stimulus response"[tiab] OR sympathetic[ti] OR "theta waves"[tiab] OR "Theta Rhythm"[Majr] OR "theta rhythm"[tiab] OR "Vagus Nerve"[Majr] OR "vagus nerve"[tiab])	5162231

3	("Clinical Study"[Publication Type] OR "Clinical Trial"[Publication Type] OR "Comparative Study"[Publication Type] OR "Controlled Clinical Trial"[Publication Type] OR "Controlled Before-After Studies"[Mesh] OR "Cross-Over Studies"[Mesh] OR "Double-Blind Method"[Mesh] OR "Empirical Research"[Mesh] OR "Non-Randomized Controlled Trials as Topic"[Mesh] OR "Control Groups"[Mesh] OR "Single-Blind Method"[Mesh] OR "Epidemiologic Research Design"[Mesh] OR pretest*[tiab] OR posttest*[tiab] OR quasi-experiment*[tiab] OR "parallel design"[tiab] OR intervention*[tiab])	5287695
4	#1 AND #2 AND #3	6813
5	#4 NOT ("Review"[Publication Type] OR "Review Literature as Topic"[Mesh] OR "Scoping Review"[Publication Type] OR "Systematic Review"[Publication Type] OR "Scoping Review as Topic"[Mesh] OR "Systematic Review as Topic"[Mesh] OR "Chronic Disease"[Mesh] OR "Noncommunicable Diseases"[Mesh] OR chronic-disease*[tiab] OR chronic-illness*[tiab] OR chronically-ill*[tiab] OR noncommunicable-disease*[tiab] OR musician*[tiab] OR musician*[tiab] OR performing-artist*[tiab] OR review*[ti])	5689
6	Filters: Abstract, English, Humans, Adult: 19-44 years, Middle Aged: 45-64 years, MEDLINE	1381

Database: Scopus
Host: Elsevier
Data Parameters: 1960 to Present
Date Searched: April 30, 2025
Searcher: Rebecca Billings

Search Strategy:

#	Searches	Results
1	(TITLE-ABS-KEY ((choice* OR favorite* OR favourite* OR individual* OR self-select* OR select* OR personal* OR prefer* OR non-prefer* OR predetermined) W/3 (beats OR genre* OR listening OR music* OR playlist* OR rhythm* OR song OR songs OR sound* OR tempo*))) AND (TITLE(alertness OR anxiet* OR arousal OR attention* OR alpha-wave* OR {alpha rhythm} OR {autonomic nervous system} OR beta-wave* OR {beta rhythm} OR {brain activity} OR brain-wave* OR {cardio vagal} OR {cognitive control} OR concentrat* OR delta-wave* OR {delta rhythm} OR electrocardiograph* OR executive-function* OR exercise OR fitness OR focus OR {heart rate} OR motor-abilit* OR motor-function* OR motor-performance* OR motor-skill* OR non-motor-function* OR {nervous system} OR {neural activation} OR neural-respons* OR parasympathetic OR perform* OR {physical exertion} OR physiology OR {psychological arousal} OR psychophysiological-outcome* OR {reaction time} OR {reaction latency} OR {response time} OR {response latency} OR regulat* OR {stimulus response} OR stress* OR sympathetic OR {theta waves} OR {theta rhythm} OR {vagus nerve})) AND PUBYEAR > 1999 AND	704

	PUBYEAR < 2026 AND (LIMIT-TO (DOCTYPE,"ar")) AND (LIMIT-TO (EXACTKEYWORD,"Human") OR LIMIT-TO (EXACTKEYWORD,"Adult") OR LIMIT-TO (EXACTKEYWORD,"Normal Human") OR LIMIT-TO (EXACTKEYWORD,"Middle Aged") OR LIMIT-TO (EXACTKEYWORD,"Article") OR EXCLUDE (EXACTKEYWORD,"Adolescent") OR EXCLUDE (EXACTKEYWORD,"Nonhuman") OR EXCLUDE (EXACTKEYWORD,"Animals") OR EXCLUDE (EXACTKEYWORD,"Animal") OR EXCLUDE (EXACTKEYWORD,"Child") OR EXCLUDE (EXACTKEYWORD,"Animal Tissue") OR EXCLUDE (EXACTKEYWORD,"Aged, 80 And Over") OR EXCLUDE (EXACTKEYWORD,"Aged")) AND (LIMIT-TO (LANGUAGE,"English")) AND (LIMIT-TO (SRCTYPE,"j"))	
2	Filters: **Include:** Journal, Articles, English, Humans, Adult, Middle-aged, normal human; 2000-present; **Exclude:** Adolescent, Nonhuman, Animal(s), Child, Animal Tissue, Aged, Aged, "80 And Over"	702

Database: Web of Science
Host: Clarivate
Data Parameters: 1990 to Present
Date Searched: April 30, 2025
Searcher: Rebecca Billings

Search Strategy:

#	Searches	Results
1	((TI=((choice* OR favorite* OR favourite* OR individual* OR self-select* OR select* OR personal* OR prefer* OR non-prefer* OR predetermined) NEAR/2 (beats OR genre* OR listening OR music* OR playlist* OR rhythm* OR song OR songs OR sound* OR tempo*))) OR AB=((choice* OR favorite* OR favourite* OR individual* OR self-select* OR select* OR personal* OR prefer* OR non-prefer* OR predetermined) NEAR/2 (beats OR genre* OR listening OR music* OR playlist* OR rhythm* OR song OR songs OR sound* OR tempo*)))	22493
2	((TI=(alertness OR anxiet* OR arousal OR attention* OR alpha-wave* OR "alpha rhythm" OR "autonomic nervous system" OR beta-wave* OR "beta rhythm" OR "brain activity" OR brain-wave* OR "cardio vagal" OR "cognitive control" OR concentrat* OR delta-wave* OR "delta rhythm" OR electrocardiograph* OR executive-function* OR exercise OR fitness OR focus OR "heart rate" OR motor-abilit* OR motor-function* OR motor-performance* OR motor-skill* OR non-motor-function* OR "nervous system" OR "neural activation" OR neural-respons* OR parasympathetic OR perform* OR "physical exertion" OR physiology OR "psychological arousal" OR psychophysiological-outcome* OR "reaction time" OR "reaction latency" OR "response time" OR "response latency" OR	3317684

	regulat* OR "stimulus response" OR stress* OR sympathetic OR "theta waves" OR "theta rhythm" OR "vagus nerve")))	
3	#1 AND #2	1952
4	Filters: 2000-2025; exclude everything but articles	
5	((TI=(musician* OR music-student* OR performing-art*)) OR AB=(musician* OR music-student* OR performing-art*)) OR AK=(musician* OR music-student* OR performing-art*)	18267
6	5 NOT 6	1504

Notes: *Searched in Web of Science Core Collection, editions included: Science Citation Index Expanded (SCI-EXPANDED) 1990-present; Social Sciences Citation Index (SSCI) 1990-present; Arts & Humanities Citation Index (AHCI) 1990-present; & Emerging Sources Citation Index (ESCI) 2018-present.*
